# Genetic Screening for EMS-Induced Maize Embryo-Specific Mutants Altered in Embryo Morphogenesis

**DOI:** 10.1534/g3.117.300293

**Published:** 2017-10-04

**Authors:** Dale C. Brunelle, Janice K. Clark, William F. Sheridan

**Affiliations:** Department of Biology, University of North Dakota, Grand Forks, North Dakota 58202

**Keywords:** maize, embryo-specific, EMS mutagenesis, morphogenesis, mutants

## Abstract

We have previously identified *embryo-specific* (*emb*) mutations that resulted in maize kernels containing abnormal embryos with normal-appearing endosperm among the progeny of active Robertson’s *Mutator* stocks. Our rationale for the mutant screen described here is that it should be possible to produce ethyl methane sulfonate (EMS)-induced *emb* mutations at a frequency higher than that obtained by transposon mutagenesis and with greater ease. This proved to be the case when we screened for mutations that are embryo-specific among progeny of materials generated with EMS-treated pollen. The EMS-induced *emb* mutation frequency reported here is nearly three times the 4.5% we obtained with the transposable element stocks. The 45 mutants reported here were all tested for germination capacity and nearly all were lethal. The embryo phenotypes of 34 mutations were examined by dissection of the mature embryos. All were found to be retarded in development and morphologically abnormal. Half of the mutants in this group were blocked in the proembryo and transition stages. They likely include mutations in nuclear genes coding for plastid proteins. The other 17 are mainly blocked in the coleoptilar stage, or in later stages with a low frequency. This group likely includes mutations in genes regulating the completion of shoot apical meristem (SAM) development and accompanying morphogenetic events. Most of the complementation tests using 19 of the mutations in 35 unique combinations complimented each other, except for two pairs of mutations with similar phenotypes. Our results provide additional evidence for the presence of many *emb* loci in the maize genome.

Early studies on maize kernel and embryo development (Demerec 1923; Mangelsdorf 1926; Wentz 1930) were performed with spontaneous mutations that affected both endosperm and embryo development. The genetic analysis of the maize kernel and its components was facilitated by the development of the use of EMS to induce mutations in maize pollen suspended in mineral oil (Neuffer and Coe 1978). This protocol was used to produce over 100 defective kernel (*dek*) mutants, most of which are lethal because of a failure to germinate, that are found to be located throughout the maize chromosomes (Neuffer and Sheridan 1980). These lethal mutants were tested for auxotrophy by culturing immature mutant embryos. During the embryo culturing experiments, some of the *dek* mutants were found to be blocked in embryo morphogenesis prior to the formation of leaf primordia and consequently incapable of germination. These observations led to a series of reports on several *dek* mutants wherein the genetic programs governing embryogenesis were lethally disturbed in different respects (Sheridan and Neuffer 1981, 1982; Clark and Sheridan 1986, 1988; Sheridan and Thorstenson 1986; Sheridan and Clark 1987).

The results obtained with the studies of the *dek* mutants cited above led to a search for maize mutations that, in contrast to the *dek* mutations, which affect both endosperm and embryo development, specifically affect embryo development. This effort resulted in the isolation of 51 *emb* mutations, representing 45 independent mutation events among self-pollinated ears of *Mutator* stocks produced by Donald S. Robertson (Clark and Sheridan 1991; Sheridan and Clark 1993). For a detailed description of maize embryo morphogenesis and these mutants see Clark (1996).

The morphological characterization of the mature embryo phenotypes of these *emb* mutants revealed that the same types of embryo blockage and abnormal morphogenesis observed in our studies of EMS-induced lethal *dek* mutants were found among the *emb* mutants obtained from Robertson’s *Mutator* stocks. Among these *dek* mutants, there were many blocked at the proembryo and transition stages, as well as mutants at abnormal coleoptilar stages that lacked any sign of forming a SAM (Sheridan and Neuffer 1981, 1982; Clark and Sheridan 1986, 1988; Sheridan and Thorstenson 1986). The presence of mutant embryos with similar abnormal morphologies in kernels lacking normal-appearing endosperm [the EMS-induced *dek* mutants of Neuffer and Sheridan (1980)] and in kernels possessing normal-appearing endosperm [the putative *Mutator*-induced *emb* mutants of Clark and Sheridan (1991) and Sheridan and Clark (1993)] was of considerable interest.

We have reasoned that it should be possible to produce EMS-induced embryo-specific mutations at a frequency equal to or higher than that obtained by transposon mutagenesis and with greater ease. This has been the rationale for the mutant screen described here. This proved to be the case when we conducted a search for mutations that are embryo-specific among progeny of materials generated with EMS-treated pollen as described herein.

## Materials and Methods

### Production of mutants

The new embryo mutations were produced by treating maize pollen with EMS (Catalog No. M0880; Sigma) suspended in mineral oil (Catalog No. M8410, light oil; Sigma). This was prepared by adding 33 μl of EMS to 100 ml of mineral oil (Neuffer 1994). The suspension was vigorously stirred and divided among three 50 ml plastic bottles. Pollen was collected in brown Kraft paper tassel bags that were set up on tassels the evening prior to the day of treatment. The pollen was passed through a fine-mesh kitchen strainer to screen out anthers and any debris; ∼7 cm^3^ of pollen was measured in a 10 ml graduated cylinder and then added to each of the plastic bottles containing the EMS/mineral oil suspension. The bottles were capped and then intermittently shaken by hand for 35, 55, or 75 min following the addition of pollen to the suspension. At the end of the selected time period, the cap on the bottle was removed, replaced with a cap that had a hole in it, and a 250 μl disposable pipette tip was securely inserted into the cap, which was prepared prior to the day of pollen treatment. The mineral oil/pollen suspension was immediately applied to silks of the recipient ears by squirting the suspension onto the silks, which had been trimmed (cut back) the previous day. The 33 ml of suspension was applied to 40–45 ears within a period of 5–10 min. The pollinated ears were immediately covered with brown Kraft pollinating bags that had been previously marked with the identity of the pollen parent and the ear parent, and were stapled around the stem of the plant. The pollinating operation was performed by a team of two persons: one applying the mineral oil/pollen suspension and the second applying the marked pollinating bags over the freshly pollinated ear shoots. A third pollinating team member continued to intermittently shake the remaining bottles of pollen suspensions and to monitor the passage of time. The contents of the other two bottles were subsequently applied to trimmed silks and covered in accordance with the planned pollinating schedule. The two individuals performing the pollinations wore hooded safety coveralls, latex gloves, and eye protection. The individual responsible for shaking the pollen suspensions and monitoring the duration of EMS treatment wore latex gloves and eye protection. Following the completion of the pollinations with the EMS-treated pollen, those areas of the experimental field containing the pollinated ears were cordoned off with yellow caution tape marking them as prohibited for entry until harvest time at ∼45–50 d following pollination. At the time of harvest, the original pollinating bags were removed and replaced with new marked bags, into which each of the pollinated ears was placed following removal from the plant and husking. The original pollinating bags were collected into black plastic trash bags that were sealed and disposed of.

### Screening of kernels for embryo mutations

Following drying on a forced-air dryer, the ears were labeled with tags marked with the ear pedigree and attached to the base of the ear with a parcel hook. The ears selected as sources of kernels for advancing to the next generation to screen for new mutations were those ears that exhibited ∼20–30% kernel set. The pollen parent source was the W22 inbred converted to the homozygous *R1-scm2* purple stock kindly provided by James Birchler or was a W22 inbred stock converted to a homozygous *r1-scm3* yellow kernel stock originally provided by Thomas Brutnell. The ear parent for the crosses with the EMS-treated pollen was an inbred B73 stock originally provided by Thomas Brutnell. The B73 inbred line is a colorless yellow stock because it is homozygous recessive at two loci where dominant alleles (*C1* and *R1*) are required for kernel color. In addition to selection of crossed ears with reduced kernel set, ears were also favored for sampling when they bore some kernels containing colorless yellow sectors on a purple aleurone background, as recommended to the corresponding author by M. Gerald Neuffer (personal communication).

A sample of 15 kernels was removed from the middle section of each selected ear and was planted in an experimental field either in Grand Forks, North Dakota or on Molokai, Hawaii. The plants grown from the 15 kernel samples were self-pollinated to produce mature ears bearing F2 kernels that were harvested 40–50 d after pollination. In preparation for screening for new mutations, a 100 kernel sample was removed from the middle section of each of the self-pollinated ears and placed in an envelope marked with the information contained on the tag attached to the source ear. These kernel samples were subsequently poured out onto a plastic tray and the kernels were turned over so that the embryo side faced upward; the faces of the kernels were scrutinized with the aid of a 2× magnifying lens mounted on a lamp containing a 60 W light bulb. When some kernels were observed to lack a fully developed embryo, the kernels were separated into two groups: the normal-appearing kernels, and the mutant kernels with normal-appearing endosperm but abnormal embryos. The number of mutant kernels was counted and recorded as a percentage value, and this was noted on the small coin envelope into which the kernels with mutant embryos were placed. The normal kernels were placed in a larger seed envelope bearing the ear pedigree, upon which the mutant segregation percent was noted. The small coin envelope containing the mutant samples was placed in the larger seed envelope, which was then filed.

### Morphological examination of mutant embryos

The extent of mutant embryo development was assayed by examination of mature kernels containing mutant embryos. A sample of 12–15 kernels was taken from the small coin envelope containing kernels with mutant embryos prepared during screening of 100 kernel samples from self-pollinated ears, as described in the preceding section. This kernel sample was placed embryo side down on moist filter paper in 120 mm diameter glass Petri dishes that were covered and sealed with Parafilm strips. The Petri dishes were kept at room temperature and, after 2–3 d, the kernels were removed and dissected with the aid of a Leica EZ4HD dissecting microscope. The overlying pericarp was removed by cutting along three sides on the kernel face overlying the embryo so as to expose it. This often involved using the scalpel blade to carefully lift away the pericarp and the underlying fibrous layer of tissues in order to clearly display the entire embryo nestled in the naturally formed cleft of the underlying endosperm tissue. The dissections were usually performed under a magnification setting of 8×, but sometimes a higher magnification was employed in order to aid in the delicate use of scalpel and fine-tip forceps to remove any tissues overlying the embryo. At least 10 mutant embryos of a mutant embryo sample were photographed using a Leica digital camera on a Leica model EZ4HD microscope. The resulting images and descriptive information, including ear pedigree, embryo stage of each mutant embryo, and magnifications of each image, were captured and noted in a computer file.

**Figure 1:**
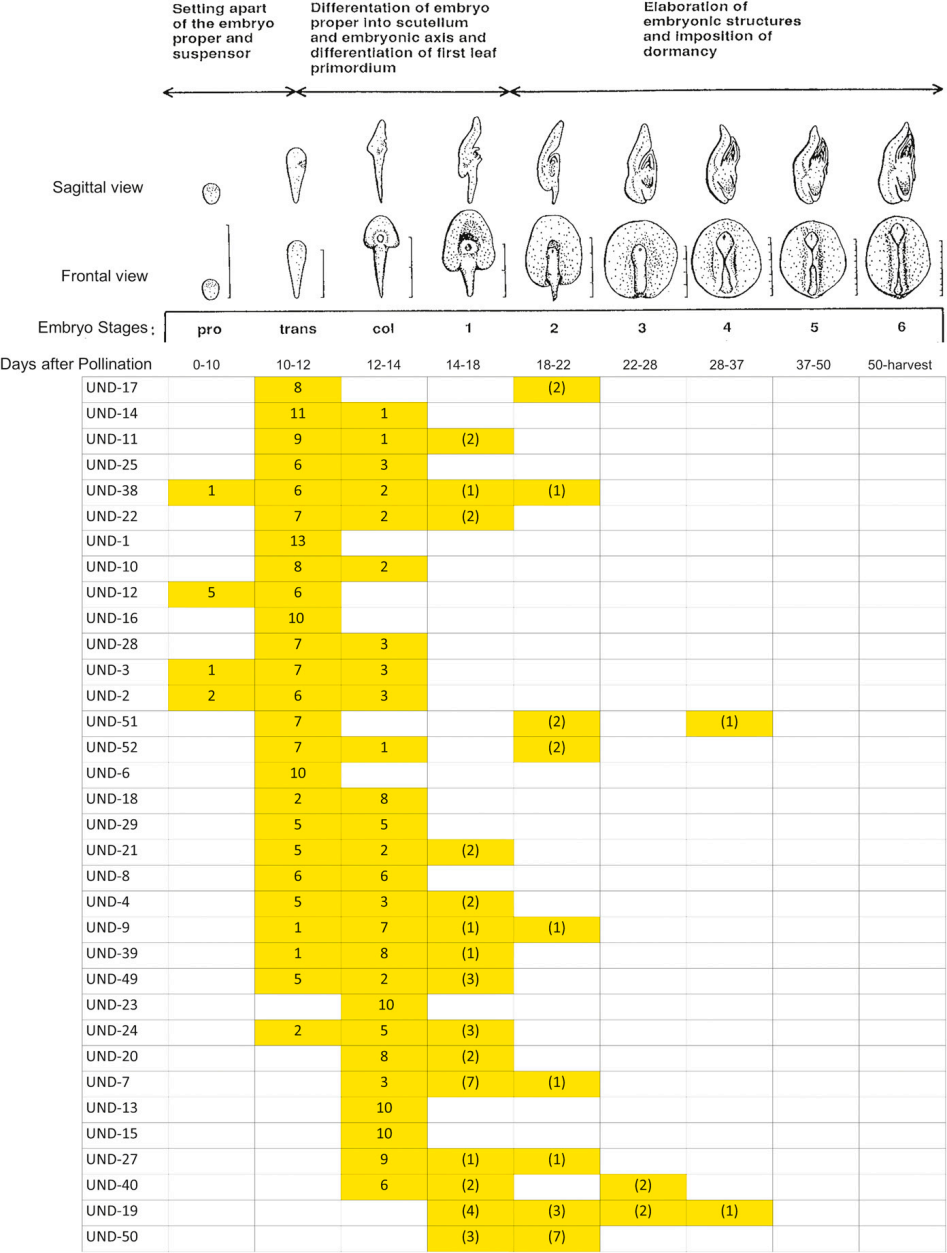
The frequency of embryos blocked at each stage is shown for each of the 34 mutants under the embryo stage heading. For stages 1–6, values in “()” indicate the number of embryos that appear morphologically farther along in development beyond the coleoptilar stage; however, we have not confirmed the development of leaf primordia. co, coleoptilar stage; pro, proembryo stage; trans, transition stage.

The stage of mutant embryo development was recorded for each embryo based on the embryo description and terminology described by Abbe and Stein (1954). In this classification scheme, the initial three stages that precede the formation of the first leaf primordium are termed the proembryo stage, the transition stage, and the coleoptilar stage. These stages are followed by stages 1–6, wherein the first through sixth leaf primordia are formed (Figure 1).

### Germination tests

In order to test the germination capacity of mutant embryos, sand bench germination tests were conducted. A single segregating self-pollinated ear was sampled for each mutant by planting 25 mature kernels containing mutant embryos and 25 kernels containing normal embryos. Germination was scored after 21 d.

### Allelism tests

Allelism was assessed by means of a complementation test in which a cross was made between two plants, using the double pollination technique described by Sheridan and Clark (1987). The first mutant was grown as a colorless (yellow) stock, while the other mutant was grown as a colored stock so that its progeny kernels were marked by anthocyanin color in the aleurone. The tests were performed by self-pollinating the ear on the colored parent plant and using its pollen to cross onto half of the silks of the colorless parent plant. The pollen of the colorless parent plant was applied to the other half of the silks of its ear to accomplish self-pollination of half of the kernels on that ear (Sheridan and Clark 1987). After the ears were harvested, dried, and tagged, the ears of the two plants used for the double pollination test were matched up. The kernels on the colored parent ear were sampled and scored for mutant embryo segregation to determine if the plant bearing them was heterozygous for the *emb* mutation. In cases where the colored parent plant was heterozygous, the kernels on the self-pollinated side of the double-pollinated ear were sampled and scored to determine if the plant bearing them was heterozygous for the second mutation. In cases where the colorless parent plant proved to be heterozygous, the kernels on the cross-pollinated side of the double-pollinated ear were sampled and scored for mutant embryo segregation. If all of these kernels displayed normal embryos, the two independently-derived mutations complemented each other and the mutations represented mutations in different genes. If, on the other hand, the kernels from the cross-pollinated side of the ear from the colorless parent plant segregated for the mutant embryo phenotype, then the two mutations failed to complement each other and the two mutations represented heritable changes in the same gene and were therefore allelic at the same locus. Converting each of the mutations into both a colorless and a colored B73 inbred stock by repeated backcrossing facilitated the allelism tests. 

### Data availability

Strains are available on request. Supplemental Material, Table S1 contains detailed descriptions of all supplemental data.

## Results

### Production and mutation frequency of embryo-specific mutations

During the summer of 2011, the crosses made with EMS-treated pollen of the W22 *r1-scm3* stock onto the same stock yielded numerous ears with reduced kernel set. These served as the source of kernels planted in the winter nursery to produce 140 plants that were self-pollinated and also crossed onto the B73 inbred stock. Screening of kernels from the self-pollinated ears identified 17 *emb* mutants, a frequency of 12.1%. During the summer of 2012, EMS-treated pollen was used to cross the W22 inbred pollen directly onto silks of the B73 inbred. Kernels from these F1 ears were planted in the subsequent winter nursery to produce 98 plants that were self-pollinated. Screening of the 98 ears from these plants identified 13 *emb* mutations, a frequency of ∼13.3%. The yield of 30 *emb* mutations from the 238 screened ears resulted in an overall mutation frequency of ∼12.6%. An additional 27 *emb* mutations were obtained using EMS-treated W22 pollen crossed onto silks of the B73 inbred, but the number of ears screened is not available as many of the nonsegregating ears were discarded because of mold on the ears. All of the *emb* mutations were propagated by planting kernels taken from the original segregating ear (the founder ear), self-pollinating the resulting plants, and crossing each onto a B73 stock. Screening of the self-pollinated ears identified those plants heterozygous for the *emb* mutation; this enabled the identification of the crossed ears that received pollen from the heterozygous plants and these ears were used to further propagate the mutations.

### Segregation frequency and lethality

The 45 mutants reported on here are listed in Table 1, where the segregation frequency of kernels with normal-appearing endosperm but mutant embryos is shown for each mutant. This frequency ranged from a low of 12.0% for UND-37 to a high value of 40.0% for UND-1, with a mean value of 22.9%. Samples of normal kernels and of kernels containing mutant embryos were taken from a single self-pollinated ear for each of the 45 mutants. The number of kernels from each of these samples that germinated is shown in Table 1. A majority (25) of the mutants exhibited no germination, while 16 of the mutants exhibited one to three germination events, and four exhibited four or more germination events. Because nearly all of the mutants were blocked prior to elaboration of leaf primordia (described below) and therefore lacked germination capacity, we attributed the germination events to be the result of the occasional escape of the mutant embryo from its developmental block or the suppression of the mutant embryo phenotype. Because nearly all of the seedlings produced by the germination of kernels with mutant embryos died at the seedling stage, it is not likely that the germination events of these kernels represent mis-scoring of embryo phenotype. The green seedlings from the germination of mutant embryos were weak plants, but were not discolored and were occasionally misshapen. All of these seedlings died at the seedling stage except for UND-52, which grew into a normal-appearing plant. Eleven of the mutants listed in Table 1 (UND-3, UND-8, UND-9, UND-18, UND-22, UND-25, UND-29, UND-38, UND-52, UND-53, and UND-56) produced one to four albino seedlings among the seedlings that germinated from normal kernels; three of these (UND-22, UND-52, and UND-56) also produced one albino seedling from the planted *emb* kernels. Additionally, although UND-28 did not produce any albino seedlings from the normal kernels planted, two albino seedlings were present among the three seedlings produced from the planted *emb* kernels. A χ^2^ test was conducted on the segregation ratio of the normal and *emb* kernels for goodness of fit to the expected 3:1 ratio for each of the 45 mutants listed. The *P*-value for each mutant is shown and only five of them (UND-1, UND-8, UND-33, UND-37, and UND-42) had a *P*-value of < 0.05; among these, only UND-8 produced any albino seedlings.

**Table 1:** Embryo-specific maize mutations

Mutation	Source Ears for Germination Tests	Percent Mutant Kernels (%)	Number of Normal Kernels	Number of *emb* Kernels	Expected Ratio	χ^2^ Test *P*-Value	Number of Normal Kernels Planted	Number of Seedlings	Number of *emb* Kernels Planted	Number of Seedlings
UND-1	KK21-3⊗	40.0	60	40	3:1	0.0005	25	21	25	0
UND-2	KK22-10⊗	19.0	81	19	3:1	0.166	25	25	25	1
UND-3	KK23-18⊗	21.0	79	21	3:1	0.356	25	25 (2w)	25	0
UND-4	KK24-8⊗	23.0	77	23	3:1	0.644	25	25	25	2
UND-6	KK25-5⊗	24.0	76	24	3:1	0.817	25	25	25	1
UND-7	KK26-18⊗	26.0	74	26	3:1	0.817	25	25	25	0
UND-8	KK59-3⊗	14.0	86	14	3:1	0.011	25	20 (4w)	25	0
UND-9	KK-60-14⊗	22.0	78	22	3:1	0.488	25	24 (1w)	25	0
UND-10	KK29-19⊗	26.0	74	26	3:1	0.817	25	25	25	1
UND-11	KK30-17⊗	26.0	74	26	3:1	0.817	25	25	25	0
UND-12	HH525-1⊗	29.0	71	29	3:1	0.356	25	20	25	0
UND-13	KK64-3⊗	24.0	76	24	3:1	0.817	25	25	25	3
UND-14	KK33-1⊗	25.0	75	25	3:1	1	25	23	25	3
UND-15	KK66-5⊗	25.0	75	25	3:1	1	25	25	25	0
UND-16	KK121-11⊗	23.0	77	23	3:1	0.644	25	24	25	0
UND-17	KK122-15⊗	27.0	73	27	3:1	0.644	25	24	25	1
UND-18	KK124-5⊗	19.0	81	19	3:1	0.166	25	25 (1w)	25	0
UND-19	JJ252-2⊗	18.0	82	18	3:1	0.106	25	24	25	0
UND-20	KK105-8⊗	24.0	76	24	3:1	0.817	25	25	25	0
UND-21	KK131-4⊗	24.0	76	24	3:1	0.817	25	25	25	0
UND-22	JJ266-12⊗	20.0	80	20	3:1	0.248	25	25 (1w)	25	1 (w)
UND-24	KK134-3⊗	25.0	75	25	3:1	1	25	24	25	3
UND-25	KK135-9⊗	23.0	77	23	3:1	0.644	25	25 (2w)	25	3
UND-26	HH545-13⊗	27.0	73	27	3:1	0.644	25	15	25	4
UND-27	HH15-7⊗	21.0	79	21	3:1	0.356	25	24	25	0
UND-28	KK39-10⊗	24.0	76	24	3:1	0.817	25	24	25	3 (2w)
UND-29	KK40-3⊗	18.0	81	18	3:1	0.118	25	25 (3w)	25	0
UND-31	KK42-11⊗	25.0	75	25	3:1	1	25	24	25	0
UND-32	HH553-2⊗	23.0	77	23	3:1	0.644	25	24	25	11
UND-33	HH21-5⊗	21.9	73	40	3:1	0.011	25	17	25	1
UND-36	HH104-11⊗	32.4	50	24	3:1	0.14	25	7	25	0
UND-37	KK123-9⊗	12.0	88	12	3:1	0.003	25	24	25	2
UND-38	HH494-2⊗	24.0	76	24	3:1	0.817	25	25 (3w)	25	0
UND-39	KK113-4⊗	26.0	74	26	3:1	0.817	25	25	25	0
UND-40	KK139-2⊗	18.0	82	18	3:1	0.106	25	24	25	3
UND-42	KK141-3⊗	14.0	86	14	3:1	0.011	25	25	25	1
UND-49	KK143-5⊗	19.0	81	19	3:1	0.166	25	25	25	0
UND-50	KK45-2⊗	18.0	82	18	3:1	0.106	25	25	25	0
UND-51	KK46-1⊗	30.0	70	30	3:1	0.248	25	25	25	0
UND-52	KK47-5⊗	18.0	82	18	3:1	0.106	25	23 (3w)	25	1 (w)
UND-53	KK48-6⊗	31.0	69	31	3:1	0.166	25	11 (3w)	25	0
UND-54	KK49-7⊗	23.0	77	23	3:1	0.644	25	23	25	0
UND-55	GG491-6⊗	19.0	81	19	3:1	0.166	25	9	25	7
UND-56	KK51-2⊗	17.0	83	17	3:1	0.065	25	19 (3w)	25	6 (1w)
UND-57	KK52-7⊗	23.0	77	23	3:1	0.644	25	23	25	0

### Extent of mutant embryo morphogenesis

In order to assess the fullest extent of mutant embryo morphogenesis, we examined the stage of mutant embryo development in mature kernels for 34 of the *emb* mutants. In some cases, the mutant embryos advanced to a normal-appearing proembryo, transition, or coleoptilar stage, or beyond, and were consistently normal in their appearance; but for a given mutant, some mutant embryos may have advanced beyond others before stoppage. The 10 or more embryos examined for each mutant were assessed as having a morphologically normal or abnormal appearance for their developmental stage, and as exhibiting uniformity or variability among the normal or abnormal-appearing embryos in their morphology. The sequence of the embryos listed is based on our assessment of the extent of advancement of the embryos from the proembryo stage, to the transition stage, and onward to the coleoptilar stage and beyond. The embryo phenotypes of the 34 mutants examined by dissection of mature kernels are presented in Figure 1.

**Figure 2:**
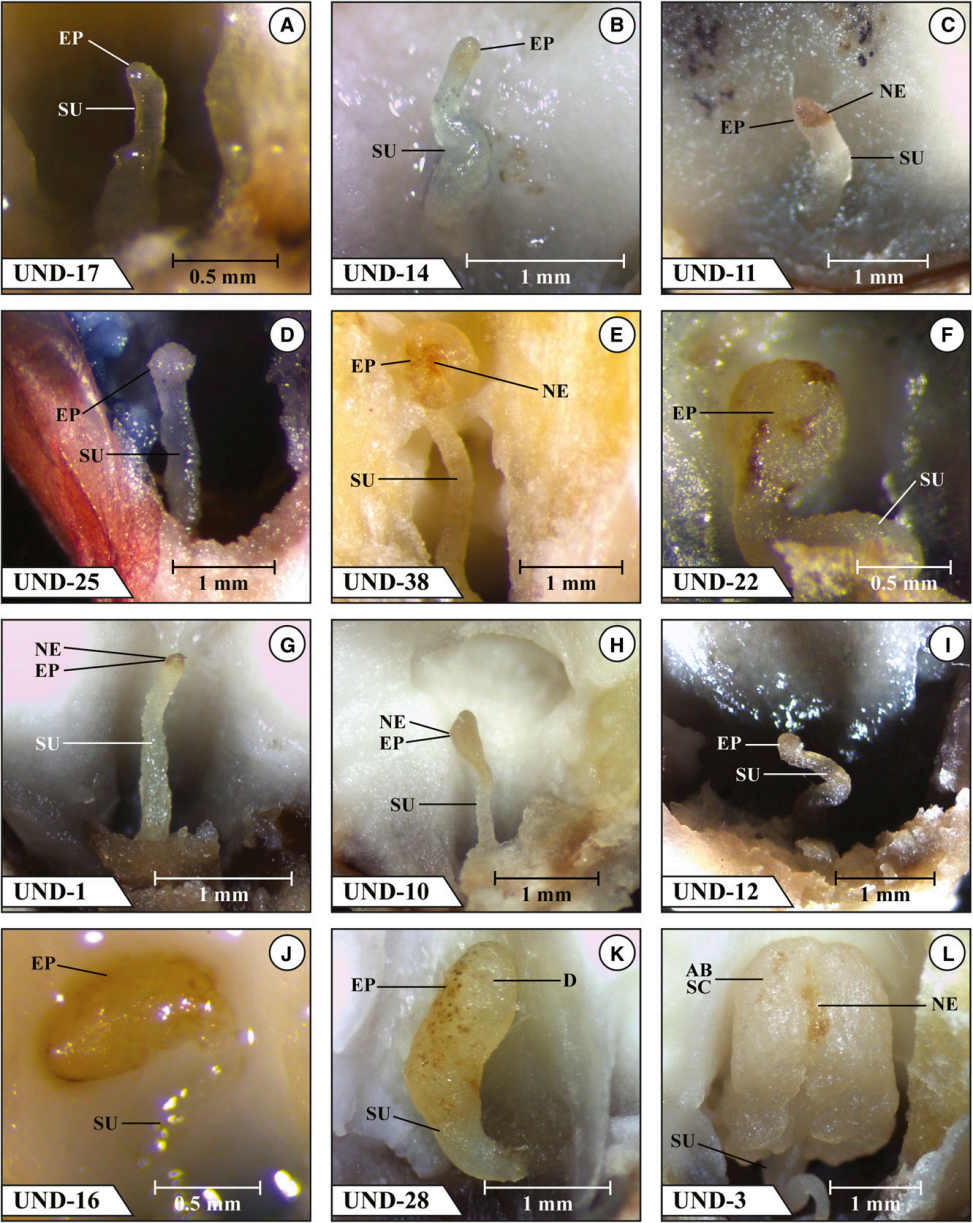
Mutant *emb* embryos in fresh dissection at kernel maturity. (a) UND-17 transition stage embryo with swollen SU; (b) UND-14 transition stage embryo with an elongated swollen SU and light tan-colored EP indicating early NE; (c) UND-11 transition stage embryo with a necrotic EP; (d) UND-25 normal-appearing transition stage embryo; (e) UND-38 abnormal transition stage embryo with early signs of NE in the EP region and an elongated SU; (f) UND-22 abnormal transition stage embryo with a swollen SU; (g) UND-1 abnormal transition stage embryo with an unexpanded EP showing NE in its uppermost region; (h) UND-10 transition stage embryo with asymmetric EP region showing early NE; (i) UND-12 transition stage embryo with a twisted SU; (j) UND-16 transition stage embryo with a swollen EP showing early NE; (k) UND-28 abnormal transition stage embryo with a swollen SU and a swollen EP region bearing a dimpled region on its upper front surface; (l) UND-3 abnormal coleoptilar stage with an abnormal SC-like structure flanking a central cleft showing NE. AB, abnormal; D, dimple; EP, embryo proper; NE, necrosis; SAM, shoot apical meristem; SC, scutellum; SU, suspensor.

The first 12 mutants listed in Figure 1 (UND-17, UND-14, UND-11, UND-25, UND-38, UND-22, UND-1, UND-10, UND-12, UND-16, UND-28, and UND-3) were mostly blocked at the transition stage, with all but three mutants (UND-1, UND-12, and UND-16) having one to three embryos blocked at an abnormal coleoptilar stage consisting of an expanded embryo proper region (the uppermost region of the transition stage embryo) lacking evidence of morphogenesis of a coleoptilar ring or SAM. Four of the 12 mutants had some embryos that appeared, on the basis of their frontal surface morphology, to have advanced beyond the coleoptilar stage; UND-17 had two embryos at stage 2, UND-11 had two embryos at stage 1, UND-38 had one embryo at stage 1 and one embryo at stage 2, and UND-22 had two embryos at stage 1. Images of a selected embryo in a dissected mature kernel that are more or less typical for each of these 12 mutants are shown in Figure 2.

**Figure 3:**
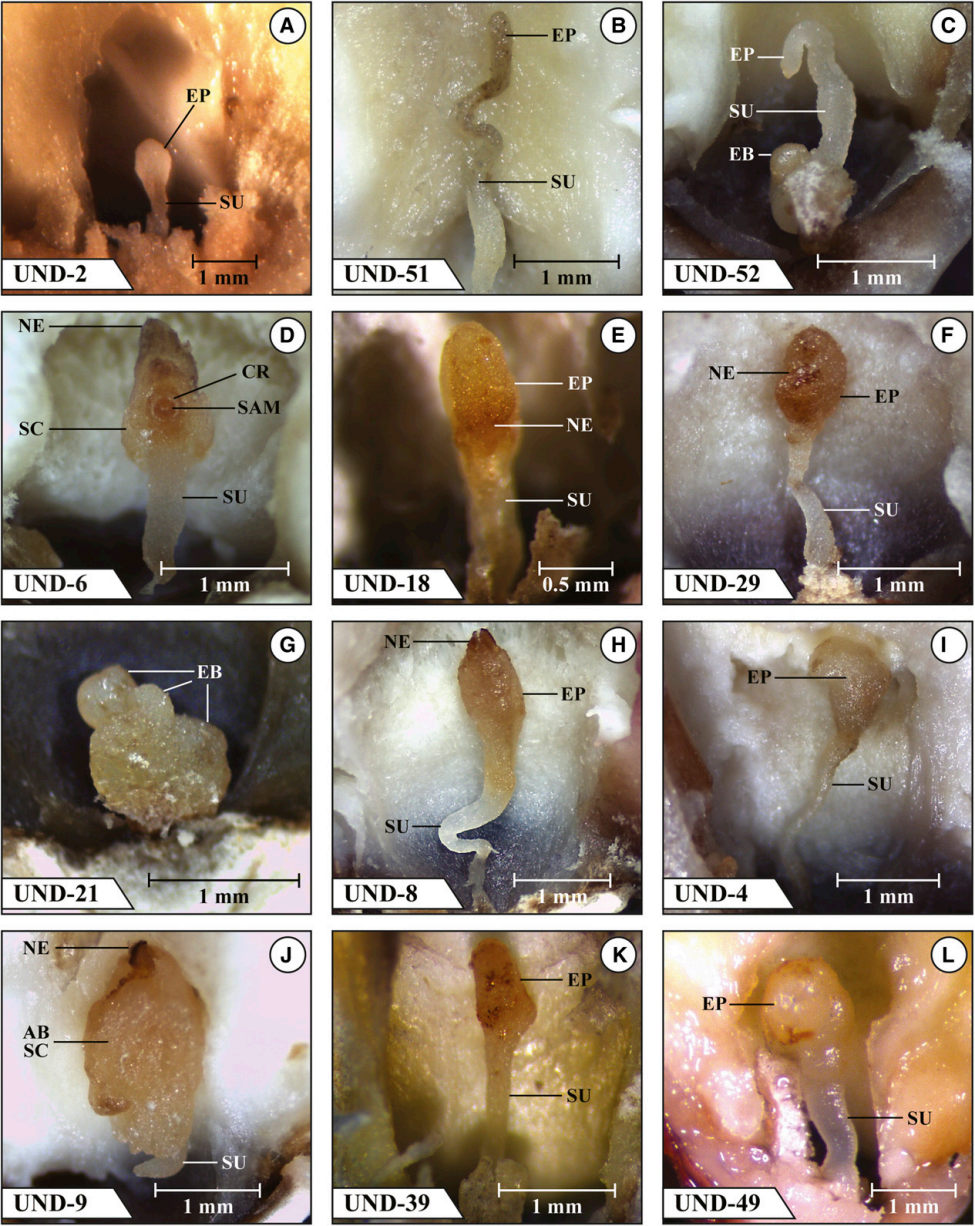
Mutant *emb* embryos in fresh dissection at kernel maturity. (a) UND-2 transition stage embryo with necrotic-appearing SU; (b) UND-51 abnormal transition stage with an elongated SU topped by necrotic-appearing elongated narrow EP region; (c) UND-52 transition stage embryo with a swollen SU and an EB at its base; (d) UND-6 late transition stage/early coleoptilar stage with a necrotic SAM nearly surrounded by a CR and unexpanded SC region showing NE in the upper most region; (e) UND-18 early transition stage embryo with NE in the region where the SU merges into the EP; (f) UND-29 transition stage embryo with a narrow healthy-appearing SU capped by a completely necrotic EP region; (g) UND-21 abnormal transition stage embryo with a truncated SU region and three EBs; (h) UND-8 abnormal transition stage with a healthy-appearing lower SU region subtending a necrotic upper region bearing a necrotic EP; (i) UND-4 abnormal transition stage embryo with a narrow elongated SU and an asymmetric EP; (j) UND-9 abnormal coleoptilar stage with a small healthy-appearing SU underlying an abnormal SC that has a frontal surface lacking an evidence of a SAM or CR but displays tan tissues at its edges indicating NE; (k) UND-39 abnormal late transition/early coleoptilar stage with normal-appearing SU merging into an asymmetric EP partially expanded; (l) UND-49 late transition/early coleoptilar stage embryo that is healthy-appearing with a partially expanded EP but no well-defined signs of a forming SAM or CR. AB, abnormal; CR, coleoptilar ring; EB, embryonic bulge; EP, embryo proper; NE, necrosis; SAM, shoot apical meristem; SC, scutellum; SU, suspensor.

The second group of 12 mutants listed in Figure 1 (UND-2, UND-51, UND-52, UND-6, UND-18, UND-29, UND-21, UND-8, UND-4, UND-9, UND-39, and UND-49) were all blocked at the transition and abnormal coleoptilar stages except for UND-6, which was blocked uniformly at the transition/coleoptilar stage, and UND-51, which had seven embryos blocked at the transition stage, two embryos blocked at stage 2, and one embryo blocked at stage 4. Six additional mutants advanced beyond an abnormal coleoptilar stage; UND-52 had two embryos reach stage 2, UND-21 had two embryos reach stage 1, UND-4 had two embryos reach stage 1, UND-9 had one embryo reach stage 1 and one embryo reach stage 2, UND-39 had one embryo reach stage 1, and UND-49 had three embryos reach stage 1. Images of a selected embryo in a mature kernel that are more or less typical for each of these 12 mutants are shown in Figure 3.

**Figure 4:**
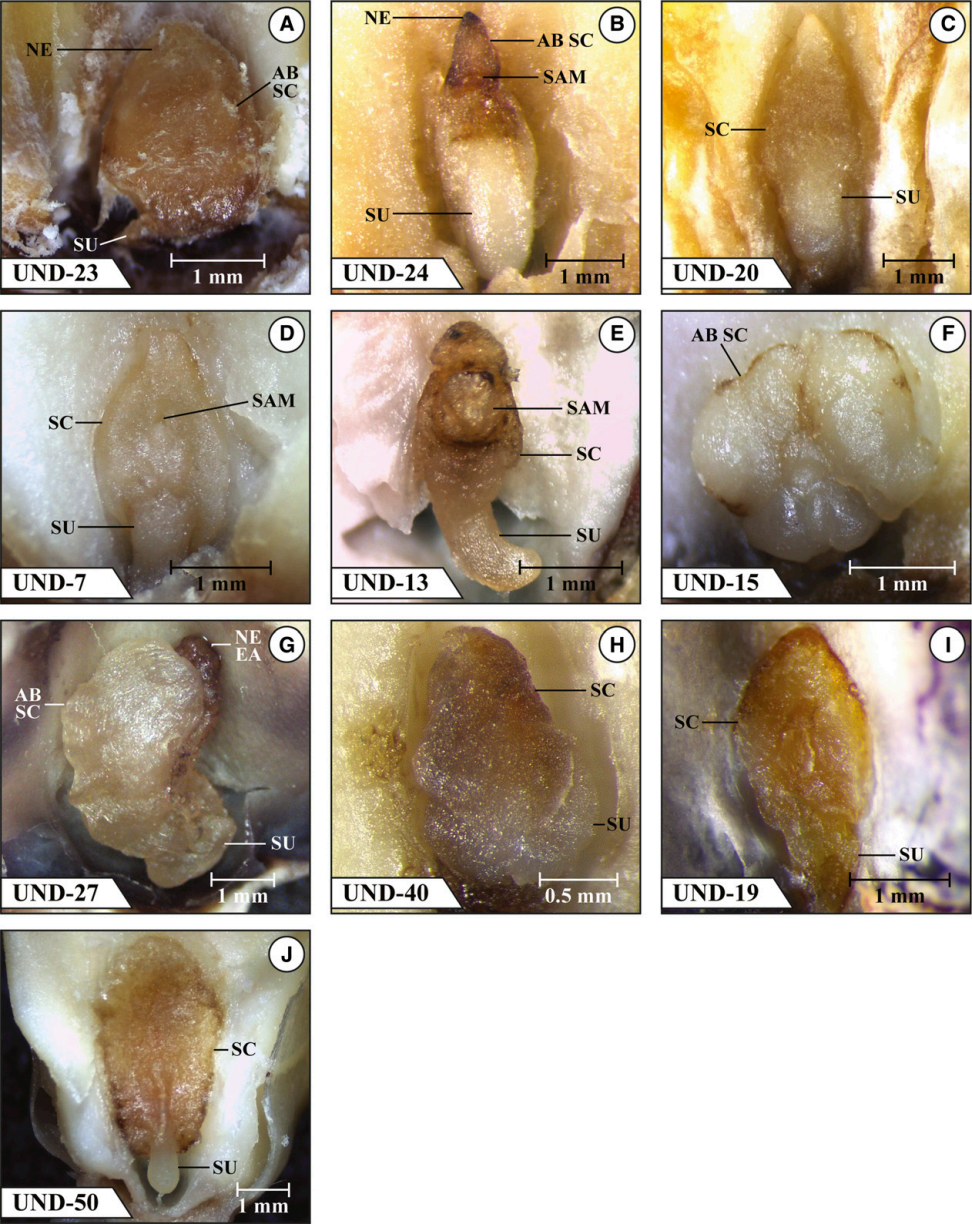
Mutant *emb* embryos in fresh dissection at kernel maturity. (a) UND-23 abnormal coleoptilar stage with an expanded smooth surface SC showing no evidence of the initiation of a SAM or CR and subtended by a poorly defined SU region; (b) UND-24 abnormal coleoptilar stage with a greatly expanded healthy-appearing SU region subtending a reduced SC region bearing a small, well defined SAM and a necrotic abnormal SC apical region; (c) UND-20 abnormal coleoptilar stage with an expanded healthy-appearing SU and a smooth-faced SC bearing no features indicating initiation of a SAM or CR; (d) UND-7 fully formed coleoptilar stage with an expanded SU and a well-formed SC bearing a very small SAM but no well-defined CR; the entire embryo is healthy-appearing; (e) UND-13 abnormal coleoptilar stage with an abnormally large SAM and SU, a much reduced SC and no evidence of a CR; (f) UND-15 abnormal coleoptilar stage embryo with grossly enlarged healthy-appearing lobes of abnormal SC-like material surrounding a poorly defined central region lacking any well-defined structures; (g) UND-27 abnormal coleoptilar stage with an abnormal expanded SC partially covering the necrotic EA and subtended by a poorly defined SU region; (h) UND-40 abnormal coleoptilar stage with a darkened SC indicating NE and displaying no evidence of the initiation of a SAM or CR and partially covering a poorly defined SU; (i) UND-19 abnormal coleoptilar stage embryo with well-developed SC with a prominent vertical crevice in the middle of the SC in the region where the EA would normally form and showing no evidence of initiation of the SAM or CR, subtended by a broad, short SU; (j) UND-50 fully formed abnormal coleoptilar stage embryo with an abnormally shaped SC that is broader in the upper region than the bottom displaying a well-defined vertically aligned narrow band of protruding tissue where the EA would normally form and showing no evidence of initiation of a SAM or CR, and subtended by a healthy-appearing normal SU. AB, abnormal; EA, embryo axis; NE, necrosis; SAM, shoot apical meristem; SC, scutellum; SU, suspensor.

The final group of 10 mutants listed in Figure 1 (UND-23, UND-24, UND-20, UND-7, UND-13, UND-15, UND-27, UND-40, UND-19, and UND-50) were mostly blocked at an abnormal coleoptilar stage and stage 1 or beyond. However, UND-23, UND-13, and UND-15 were only blocked at an abnormal coleoptilar stage, and the embryos of UND-19 and UND-50 were all blocked at stage 1 or beyond. Other than these latter two mutants, only UND-7 had more than four embryos advance beyond an abnormal coleoptilar stage. The embryos of UND-50 were rather uniform in possessing an abnormal SAM-like and coleoptilar ring-like structure and misshapen scutellum, and were the most advanced in development among the 34 mutants examined. Images of a selected embryo in a mature kernel that are more or less typical for each of these 10 mutants are shown in Figure 4.

### The majority of the mutants tested complemented each other

Complementation tests were conducted using 19 of the *emb* mutations. Sixteen of the mutants, UND-1, UND-3, UND-4, UND-6, UND-8, UND-9, UND-14, UND-18, UND-19, UND-20, UND-21, UND-22, UND-25, UND-39, UND-49, and UND-52 were used as pollen parents and were marked by having colored aleurone kernels. Seventeen of the mutants, UND-1, UND-4, UND-6, UND-7, UND-8, UND-9, UND-10, UND-14, UND-18, UND-20, UND-21, UND-22, UND-25, UND-39, UND-49, UND-51, and UND-52 were used as ear parents and were in colorless (yellow) kernel stocks. A total of 329 double-pollinated ears were produced in the 2015–2016 and 2016–2017 winter nurseries.

In order for a complementation test to provide an unambiguous result, both the pollen parent plant and the ear parent plant must be heterozygous for the mutations being tested, and both plants must yield ears bearing a sufficient number of scoreable kernels. Among the 329 double-pollinated ears produced, 235 ears failed to meet these criteria. Among these 235 ears, 67 of the pollen parent ears did not segregate for the *emb* mutant, 26 of the pollen parent plants failed to produce a scoreable ear, 101 of the female parent plant ears did not segregate on the selfed side of the ears, and 41 of the female parent plants failed to produce a scoreable ear.

The 94 double-pollinated ears that provided unambiguous results are listed in Table S1. There were 45 combinations of the colored pollen parent and the colorless female parent (Table 2). Among these 45, there were 10 cases involving reciprocal crosses. Among the 35 unique combinations of crosses of the mutants, 33 combinations demonstrated complementation as the kernels on the crossed side of the double-pollinated ear failed to segregate for an *emb* phenotype, even though both the pollen parent self-pollinated ear and the selfed side of the double-pollinated ear segregated their respective *emb* phenotypes. All four double-pollinated ears produced by crossing UND-1 onto UND-10 (crosses 8, 9, 10, and 11) resulted in segregation for the emb phenotype among kernels on the crossed side of these ears, thereby demonstrating allelism between UND-1 and UND-10 because of the failure to complement in these crosses. Both of the double-pollinated ears produced by crossing UND-4 onto UND-9 (crosses 29 and 30) resulted in segregation for the emb phenotype among kernels on the crossed side of these ears, thereby demonstrating allelism between UND-4 and UND-9 because of the failure of complementation in these crosses. The results of the complementation tests are summarized in Table 2.

**Table 2:** Summary of the tests for complementation

Pollen Parent	UND-1	UND-3	UND-4	UND-6	UND-8	UND-9	UND-14	UND-18	UND-19	UND-20	UND-21	UND-22	UND-25	UND-39	UND-49	UND-52
Ear parent																
UND-1																C (89)
UND-4	C (1), C (2)	C (17)	NA	C (31), C (32), C (33)	C (48)	**A** (29), **A** (30)										
UND-6	C (3), C (4), C (5)		C (22), C (23)	NA	**C** (37), **C** (38), **C** (39), **C** (40)	**C** (41), **C** (42), **C** (43), **C** (44), **C** (45)										
UND-7	C (6)	C (18)	C (24), C (25)	C (34), C (35), C (36)	C (49)	**C** (24), **C** (25)										
UND-8	C (7), **C** (52)	C (19)	C (26), C (27), C (28)	C (37), C (38), C (39), C (40)	NA	**C** (50), **C** (51)										
UND-9	**C** (1), **C** (2)	**C** (17)	A (29), A (30)	C (41), C (42), C (43), C (44), C (45)	C (50), C (51)	NA									C (87), C (88)	
UND-10	A (8), A (9), A (10), A (11)	C (20), C (21)	**C** (1), **C** (2)	C (46), C (47)	C (52), **C** (7)	**C** (1, **C** (2)										
UND-14	C (12), C (13), C (14)															C (90), C (91), C (92)
UND-18										C (68)	C (74), C (75), C (76)					
UND-20								C (56), C (57)			C (77), C (78)			C (84), C (85)		
UND-21								C (58), C (59), C (60), C (61), C (62)		C (69), C (70), C (71), C (72)				C (86)		
UND-22								C (63), C (64)	C (67)			NA	C (83)			
UND-25												C (82)				
UND-39								C (65), C (66)		C (73)	C (79), C (80), C (81)					
UND-49						C (53), C (54)										
UND-51	C (15)															C (93), C (94)
UND-52	C (16)						C (55)									

Crosses between *emb* mutants that complemented each other are indicated by the symbol C, and crosses between mutants that did not complement each other are indicated by the symbol A because these mutants appear to be in the same gene and are therefore allelic. The number of symbols in each “box” of the table indicates the number of replicas of that cross. The numbers in parentheses next to each symbol correspond to the corresponding cross with its detailed results shown in Table S1. The entries in bold are based on the result observed with the reciprocal cross. For example, when UND-4 was crossed onto UND-9 (crosses 29 and 30), allelism was inferred and this is indicated in the box in the table for the cross of UND-4 as the pollen parent onto UND-9 as the ear parent, and is also shown in bold in the box in the table for UND-9 as the pollen parent and UND-4 as the ear parent, although this latter cross was not performed. Some entries enclosed in parenthesis reflect the allelism observed for UND-1 and UND-10 as well as UND-4 and UND-9. NA, not applicable.

## Discussion

### Comparison with previous screening for emb mutations

The relatively high value of 12.6% mutation frequency of *emb* mutations by chemical mutagenesis that we report here suggests that there are many gene loci whose specific functions are required for normal maize embryo development. Because we have screened for these mutations based on their selective impairment of embryo development in kernels displaying little or no readily observed defects in the endosperm development of intact kernels, we believe their analysis may be especially rewarding in seeking to identify maize genes that play essential roles in embryo morphogenesis. The EMS-induced mutation frequency reported here is nearly three times the 4.5% we obtained with the transposable element stocks. The higher mutation frequency obtained with EMS may be because it enables an unbiased screen and allows for mutations across the genome (Winston and Koshland 2016), whereas the transposable element systems require transposition events and some basis for their target sites. In any case, a large number of *emb* mutations can be readily produced. The major impediment to identifying and propagating the *emb* mutations is the fact that they cannot be readily identified by simply screening the kernels of intact ears, as can be easily done when searching for *dek* mutations. It is because the detection of *emb* mutations requires the removal of kernels from self-pollinated ears and the examination of their germinal (embryo) side, usually with some low-power magnification, to discern the presence of kernels with a normal-appearing endosperm and a reduced-size embryo.

The average segregation frequency on self-pollinated ears of 22.9% of kernels with normal-appearing endosperm but mutant embryos indicates that these mutations are, on average, segregating as expected for single-gene Mendelian factors, but also that there does not appear to be a widespread reduction in mutation transmission through either the female or male gametophyte generation. The low germination frequency of most of the mutants tested indicates that most are lethal mutations, and that they identify genes that play essential roles in embryo development.

The allelism test results reported here showed complementation for most of the 35 unique combinations tested. These tests did not involve random crossing among the *emb* mutations because we used the mutants that we had available in both colored and noncolored kernel stocks. We did select several combinations of crosses to test between pairs of mutants with similar phenotypes. Therefore, the discovery that two pairs of *emb* mutations failed to complement and are therefore allelic is not entirely an unbiased result. However, the fact that 33 of the 35 combinations tested did complement, taken together with the relatively high mutation frequency approaching that of *dek* mutations, suggest that there are hundreds of loci that can mutate to produce an emb phenotype.

## 

## Supplementary Material

Supplemental material is available online at www.g3journal.org/lookup/suppl/doi:10.1534/g3.117.300293/-/DC1.
