# Supplementary material for: Genetic Screening for EMS-Induced Maize Embryo-Specific Mutants Altered in Embryo Morphogenesis
Source: G3 (Bethesda). 2017 Oct 4;7(11):3559–70. doi: 10.1534/g3.117.300293 (PMC5677168; doi:10.1534/g3.117.300293)
Supplement: Supplementary file 1 [file 3559TableS1.docx]

Table S1. Complementation Tests- Detailed Results

| Ear No. | Pollen Parent  Selfed-colored | | Ear Parent  Selfed side-colorless | | Crossed side-colored | | Complement | |
| --- | --- | --- | --- | --- | --- | --- | --- | --- |
| 1 | UND-1 | Seg 21.0% | UND-4 | Seg 23.7% | UND-4xUND-1 | LL676-11x671-12 | No seg | Yes |
| 2 | UND-1 | Seg 21.0% | UND-4 | Seg 18.0% | UND-4xUND-1 | LL677-7x671-12 | No Seg | Yes |
| 3 | UND-1 | Seg 21.0% | UND-6 | Seg 28.6% | UND-6xUND-1 | LL678-5x671-12 | No Seg | Yes |
| 4 | UND-1 | Seg 31.0% | UND-6 | Seg 24.0% | UND-6xUND-1 | LL678-6x671-8 | No Seg | Yes |
| 5 | UND-1 | Seg 31.0% | UND-6 | Seg 30.0% | UND-6xUND-1 | LL678-7x671-8 | No Seg | Yes |
| 6 | UND-1 | Seg 22.0% | UND-7 | Seg 30.0% | UND-7xUND-1 | LL680-6x671-6 | No Seg | Yes |
| 7 | UND-1 | Seg 31.0% | UND-8 | Seg 23.8% | UND-8xUND-1 | LL682-7x671-8 | No Seg | Yes |
| 8 | UND-1 | Seg 28.0% | UND-10 | Seg 31.0% | UND-10xUND-1 | LL686-2x671-5 | Seg 25.0% | No |
| 9 | UND-1 | Seg 22.4% | UND-10 | Seg 25.6% | UND-10xUND-1 | LL686-5x671-14 | Seg 29.6% | No |
| 10 | UND-1 | Seg 28.0% | UND-10 | Seg 24.0% | UND-10xUND-1 | LL686-10x671-5 | Seg 32.9% | No |
| 11 | UND-1 | Seg 22.3% | UND-10 | Seg 29.0% | UND-10xUND-1 | LL686-12x671-7 | Seg 29.0% | No |
| 12 | UND-1 | Seg 24.0% | UND-14 | Seg 32.0% | UND-14XUND-1 | MM567-3X558-1 | No seg | Yes |
| 13 | UND-1 | Seg 24.0% | UND-14 | Seg 20.0% | UND-14XUND-1 | MM567-4X558-1 | No seg | Yes |
| 14 | UND-1 | Seg 24.0% | UND-14 | Seg 26.0% | UND-14XUND-1 | MM567-13X558-1 | No seg | Yes |
| 15 | UND-1 | Seg 28.7% | UND-51 | Seg 21.3% | UND-51XUND-1 | MM574-6X557-4 | No seg | Yes |
| 16 | UND-1 | Seg 30.4% | UND-52 | Seg 18.9% | UND-52XUND-1 | MM577-7X558-6 | No seg | Yes |
| 17 | UND-3 | Seg 27.7% | UND-4 | Seg 26.0% | UND-4xUND-3 | LL677-10x672-4 | No Seg | Yes |
| 18 | UND-3 | Seg 21.9% | UND-7 | Seg 24.0% | UND-7xUND-3 | LL681-1x672-13 | No Seg | Yes |
| 19 | UND-3 | Seg 21.9% | UND-8 | Seg 20.8% | UND-8xUND-3 | LL683-4x672-13 | No Seg | Yes |
| 20 | UND-3 | Seg 21.3% | UND-10 | Seg 20.0% | UND-10xUND-3 | LL687-3x672-7 | No Seg | Yes |
| 21 | UND-3 | Seg 17.1% | UND-10 | Seg 30.0% | UND-10xUND-3 | LL687-6x672-11 | No Seg | Yes |
| 22 | UND-4 | Seg 20.0% | UND-6 | Seg 25.0% | UND-6xUND-4 | LL697-2x710-4 | No Seg | Yes |
| 23 | UND-4 | Seg 21.0% | UND-6 | Seg 26.7% | UND-6xUND-4 | LL695-2x710-3 | No Seg | Yes |
| 24 | UND-4 | Seg 24.0% | UND-7 | Seg 25.3% | UND-7xUND-4 | LL700-6x711-11 | No Seg | Yes |
| 25 | UND-4 | Seg 20.7% | UND-7 | Seg 23.1% | UND-7xUND-4 | LL700-10x712-7 | No Seg | Yes |
| 26 | UND-4 | Seg 24.8% | UND-8 | Seg 19.6% | UND-8xUND-4 | LL702-3x712-8 | No Seg | Yes |
| 27 | UND-4 | Seg 24.8% | UND-8 | Seg 28.6% | UND-8xUND-4 | LL703-3x712-8 | No Seg | Yes |
| 28 | UND-4 | Seg 20.0% | UND-8 | Seg 23.5% | UND-8xUND-4 | LL703-12x710-4 | No Seg | Yes |
| 29 | UND-4 | Seg 20.7% | UND-9 | Seg 20.0% | UND-9xUND-4 | LL705-1x712-7 | Seg 27.5% | No |
| 30 | UND-4 | Seg 13.1% | UND-9 | Seg 26.0% | UND-9xUND-4 | LL707-9x712-1 | Seg 27.7% | No |
| 31 | UND-6 | Seg 19.0% | UND-4 | Seg 24.0% | UND-4xUND-6 | LL692-14x715-7 | No Seg | Yes |
| 32 | UND-6 | Seg 25.0% | UND-4 | Seg 26.0% | UND-4xUND-6 | LL692-8x713-1 | No Seg | Yes |
| 33 | UND-6 | Seg 29.6% | UND-4 | Seg 23.5% | UND-4xUND-6 | LL692-2x713-5 | No Seg | Yes |
| 34 | UND-6 | Seg 15.0% | UND-7 | Seg 25.0% | UND-7xUND-6 | LL699-8x715-1 | No Seg | Yes |
| 35 | UND-6 | Seg 26.0% | UND-7 | Seg 20.5% | UND-7xUND-6 | LL699-10x714-8 | No Seg | Yes |
| 36 | UND-6 | Seg 32.8% | UND-7 | Seg 29.1% | UND-7xUND-6 | LL700-9x715-2 | No Seg | Yes |
| 37 | UND-6 | Seg 19.0% | UND-8 | Seg 34.9% | UND-8xUND-6 | LL703-6x715-7 | No Seg | Yes |
| 38 | UND-6 | Seg 26.0% | UND-8 | Seg 31.9% | UND-8xUND-6 | LL703-14x714-8 | No Seg | Yes |
| 39 | UND-6 | Seg 31.1% | UND-8 | Seg 24.0% | UND-8xUND-6 | LL704-6x713-6 | No Seg | Yes |
| 40 | UND-6 | Seg 19.0% | UND-8 | Seg 32.6% | UND-8xUND-6 | LL704-7x715-7 | No Seg | Yes |
| 41 | UND-6 | Seg 31.1% | UND-9 | Seg 28.4% | UND-9xUND-6 | LL705-3x713-6 | No Seg | Yes |
| 42 | UND-6 | Seg 29.6% | UND-9 | Seg 26.7% | UND-9xUND-6 | LL705-4x713-5 | No Seg | Yes |
| 43 | UND-6 | Seg 17.0% | UND-9 | Seg 19.0% | UND-9xUND-6 | LL705-5x713-8 | No Seg | Yes |
| 44 | UND-6 | Seg 24.8% | UND-9 | Seg 19.6% | UND-9xUND-6 | LL706-10x713-9 | No Seg | Yes |
| 45 | UND-6 | Seg 31.1% | UND-9 | Seg 16.7% | UND-9xUND-6 | LL706-12x713-6 | No Seg | Yes |
| 46 | UND-6 | Seg 17.0% | UND-10 | Seg 27.6% | UND-10xUND-6 | LL708-5x713-8 | No Seg | Yes |
| 47 | UND-6 | Seg 33.0% | UND-10 | Seg 33.3% | UND-10xUND-6 | LL708-12x713-3 | No Seg | Yes |
| 48 | UND-8 | Seg 33.6% | UND-4 | Seg 26.2% | UND-4xUND-8 | LL692-7x716-2 | No Seg | Yes |
| 49 | UND-8 | Seg 19.5% | UND-7 | Seg 29.6% | UND-7xUND-8 | LL700-8x716-1 | No Seg | Yes |
| 50 | UND-8 | Seg 33.6% | UND-9 | Seg 28.0% | UND-9xUND-8 | LL706-9x716-2 | No Seg | Yes |
| 51 | UND-8 | Seg 33.6% | UND-9 | Seg 19.5% | UND-9xUND-8 | LL706-11X716-2 | No Seg | Yes |
| 52 | UND-8 | Seg 19.5% | UND-10 | Seg 33.3% | UND-10xUND-8 | LL709-6x716-1 | No Seg | Yes |
| 53 | UND-9 | Seg 27.0% | UND-49 | Seg 24.6% | UND-49xUND-9 | LL931-13x929-2 | No Seg | Yes |
| 54 | UND-9 | Seg 18.0% | UND-49 | Seg 19.5% | UND-49xUND-9 | LL931-10x929-8 | No Seg | Yes |
| 55 | UND-14 | Seg 17.4% | UND-52 | Seg 26.1% | UND-52XUND-14 | MM577-2X560-14 | No seg | Yes |
| 56 | UND-18 | Seg 25.0% | UND-20 | Seg 24.5% | UND-20XUND-18 | MM536-2X525-5 | No seg | Yes |
| 57 | UND-18 | Seg 17.1% | UND-20 | Seg 25.9% | UND-20XUND-18 | MM537-1X523-2 | No seg | Yes |
| 58 | UND-18 | Seg 23.1% | UND-21 | Seg 35.1% | UND-21XUND-18 | MM538-1X523-4 | No seg | Yes |
| 59 | UND-18 | Seg 23.1% | UND-21 | Seg 31.4% | UND-21XUND-18 | MM538-2X523-4 | No seg | Yes |
| 60 | UND-18 | Seg 25.1% | UND-21 | Seg 26.2% | UND-21XUND-18 | MM538-3X523-5 | No seg | Yes |
| 61 | UND-18 | Seg 25.4% | UND-21 | Seg 30.6% | UND-21XUND-18 | MM540-1X522-5 | No seg | Yes |
| 62 | UND-18 | Seg 25.4% | UND-21 | Seg 29.3% | UND-21XUND-18 | MM540-11X522-5 | No seg | Yes |
| 63 | UND-18 | Seg 16.0% | UND-22 | Seg 26.3% | UND-22xUND-18 | KK687-8x680-1 | No Seg | Yes |
| 64 | UND-18 | Seg 16.0% | UND-22 | Seg 20.0% | UND-22xUND-18 | KK688-1x680-1 | No Seg | Yes |
| 65 | UND-18 | Seg 17.9% | UND-39 | Seg 20.0% | UND-39XUND-18 | MM543-7X523-7 | No seg | Yes |
| 66 | UND-18 | Seg 17.1% | UND-39 | Seg 25.5% | UND-39XUND-18 | MM545-4X523-2 | No seg | Yes |
| 67 | UND-19 | Seg 18.7% | UND-22 | Seg 16.8% | UND-22xUND-19 | MM688-11x681-6 | No Seg | Yes |
| 68 | UND-20 | Seg 20.7% | UND-18 | Seg 23.1% | UND-18xUND-20 | MM530-4x525-1 | No seg | Yes |
| 69 | UND-20 | Seg 28.0% | UND-21 | Seg 17.8% | UND-21XUND-21 | MM539-4X524-3 | no seg | Yes |
| 70 | UND-20 | Seg 22.4% | UND-21 | Seg 27.0% | UND-21XUND-20 | MM540-2X525-7 | No seg | Yes |
| 71 | UND-20 | Seg 32.7% | UND-21 | Seg 28.2% | UND-21XUND-20 | MM540-6X525-2 | No seg | Yes |
| 72 | UND-20 | Seg 22.4% | UND-21 | Seg 23.3% | UND-21XUND-20 | MM540-9X525-7 | No seg | Yes |
| 73 | UND-20 | Seg 32.7% | UND-39 | Seg 19.8% | UND-39XUND-20 | MM545-6X525-2 | No seg | Yes |
| 74 | UND-21 | Seg 18.8% | UND-18 | Seg 25.0% | UND-18XUND-21 | MM530-1X526-8 | No seg | Yes |
| 75 | UND-21 | Seg 18.9% | UND-18 | Seg 22.7% | UND-18XUND-21 | MM530-3X526-3 | No seg | Yes |
| 76 | UND-21 | Seg 14.0% | UND-18 | Seg 25.0% | UND-18-UND-21 | MM530-9X527-12 | No seg | Yes |
| 77 | UND-21 | Seg 27.0% | UND-20 | Seg 16.9% | UND-20XUND-21 | MM537-6X527-14 | No seg | Yes |
| 78 | UND-21 | Seg 27.8% | UND-20 | Seg 32.8% | UND-20XUND-21 | MM537-9X527-3 | No seg | Yes |
| 79 | UND-21 | Seg 15.0% | UND-39 | Seg 22.2% | UND-39XUND-21 | MM543-8X526-5 | No seg | Yes |
| 80 | UND-21 | Seg 14.0% | UND-39 | Seg 25.3% | UND-39XUND-21 | MM543-11X524-12 | No seg | Yes |
| 81 | UND-21 | Seg 27.0% | UND-39 | Seg 32.0% | UND-39XUND-21 | MM543-12X527-14 | No seg | Yes |
| 82 | UND-22 | Seg 19.0% | UND-25 | Seg 20.0% | UND-25xUND-22 | KK690-1x682-5 | No Seg | Yes |
| 83 | UND-25 | Seg 19.3% | UND-22 | Seg 14.8% | UND-22xUND-25 | KK687-10x683-9 | No Seg | Yes |
| 84 | UND-39 | Seg 22.5% | UND-20 | Seg 30.6% | UND-20XUND-39 | MM536-3X528-3 | No seg | Yes |
| 85 | UND-39 | Seg 20.0% | UND-20 | Seg 25.0% | UND-20XUND-39 | MM536-10X528-6 | No seg | Yes |
| 86 | UND-39 | Seg 23.0% | UND-21 | Seg 39.0% | UND-21XUND-39 | MM541-4X529-6 | No seg | Yes |
| 87 | UND-49 | Seg 24.0% | UND-9 | Seg 21.6% | UND-9xUND-49 | LL932-7x930-1 | No Seg | Yes |
| 88 | UND-49 | Seg 29.0% | UND-9 | Seg 22.0% | UND-9xUND-49 | LL932-3x930-6 | No Seg | Yes |
| 89 | UND-52 | Seg 29.0% | UND-1 | Seg 22.0% | UND-1XUND-52 | MM566-12X562-9 | No seg | Yes |
| 90 | UND-52 | Seg 31.0% | UND-14 | Seg 16.0% | UND-14XUND-52 | MM568-1X562-2 | No seg | Yes |
| 91 | UND-52 | Seg 31.0% | UND-14 | Seg 18.1% | UND-14XUND-52 | MM568-8X562-2 | No seg | Yes |
| 92 | UND-52 | Seg 25.0% | UND-14 | Seg 19.2% | UND-14XUND-52 | MM570-5X562-4 | No seg | Yes |
| 93 | UND-52 | Seg 29.0% | UND-51 | Seg 40.7% | UND-51XUND-52 | MM573-1X562-7 | No seg | Yes |
| 94 | UND-52 | Seg 29.1% | UND-51 | Seg 33.0% | UND-51XUND-52 | MM573-6X562-13 | No seg | Yes |
